# First record of Phlebotomine sandflies (Diptera: Psychodidae) in the Comoros Archipelago with description of *Sergentomyia (Vattieromyia) pessoni* n. sp. and *S. (rondanomyia) goodmani comorensis* n. ssp.

**DOI:** 10.1051/parasite/2012193195

**Published:** 2012-08-15

**Authors:** F.J. Randrianambinintsoa, J. Depaquit, C. Brengues, C. Dhondt, I. Yahaya, A. Ouledi, N. Léger, V. Robert

**Affiliations:** 1 Département de Biologie Animale, Faculté des Sciences, Université d’Antananarivo Madagascar; 2 Université de Reims Champagne-Ardenne, ANSES, JE2533 – USC “Transmission vectorielle et épidémiosurveillance de maladies parasitaires (VECPAR)” 51, rue Cognacq-Jay 51096 Reims Cedex France; 3 MIVEGEC, UMR IRD 224-CNRS 5290-UM1-UM2 911, avenue Agropolis BP 64501 34394 Montpellier Cedex 5 France; 4 CNDRS BP 169 Moroni Union des Comores; 5 Coordonnateur des Programmes de recherches, Université des Comores route de la Corniche Moroni Union des Comores; 6 63, avenue Pierre Sémard 94210 La Varenne Saint Hilaire France

**Keywords:** Phlebotomine sandfly, *Sergentomyia*, DNA barcoding, taxonomy, inventory, Comoros, Mayotte, Indian Ocean, phlébotome, *Sergentomyia*, séquençage d’ADN, taxonomie, inventaire, Comores, Mayotte, Océan Indien

## Abstract

No Phlebotomine sandflies had ever been reported in the Comoros Archipelago, including the three islands of the Republic of the Union of Comoros (Grande Comore, Mohéli and Anjouan) and the French oversea department of Mayotte. During three field surveys carried out in 2003, 2007 and 2011, we provided the first record of Phlebotomine sandflies in this area. A total of 85 specimens belonging to three species were caught: a new species *S. (Vattieromyia) pessoni* n. sp. (two females from Grande Comore), a new subspecies of *Sergentomyia (Rondanomyia) goodmani* (80 specimens from Grande Comore and one from Anjouan) and *Grassomyia* sp. (two females from Mohéli). The individualisation of these taxa was inferred both from morphological criteria and sequencing of a part of the cytochrome b of the mitochondrial DNA. These taxa are closely related to Malagasy sandflies.

## Abstract (fr)

Aucun phlébotome n’avait jamais été rapporté dans l’archipel des Comores, incluant les trois îles de l’Union des Comores (la Grande Comore, Mohéli et Anjouan) et le département français d’outremer, Mayotte. Au cours de trois campagnes d’études sur le terrain en 2003, 2007 et 2011, nous avons fait la première observation de phlébotomes dans cette zone. 85 spécimens appartenant à trois espèces ont été capturés : une nouvelle espèce, *S. (Vattieromyia) pessoni* n. sp. (deux femelles de la Grande Comore), une nouvelle sous-espèce de *Sergentomyia (Rondanomyia) goodmani* (80 spécimens de la Grande Comore et une d’Anjouan) et *Grassomyia* sp. (deux femelles à Mohéli). L’individualisation de ces taxons s’est faite à la fois à partir de critères morphologiques et du séquençage d’une partie du cytochrome b de l’ADN mitochondrial. Ces taxons sont très proches des phlébotomes de Madagascar.

## Introduction

To our knowledge, no record of Phlebotomine sandfly exists in the literature and there was no case of leishmaniasis in the Comoros Archipelago. The latter includes the Islands of the Union of the Comoros (Grande Comore, Anjouan and Mohéli) and Mayotte (a French oversea department). In the neighbouring countries, little is known about Phlebotomine sandflies from South-eastern Africa (Abonnenc, 1972; Artemiev, 1985; Davidson, 1987) and from Madagascar (Depaquit *et al.*, 2002, 2004a & b , 2007, 2008; Léger *et al.*, 2005). We performed three field works in order to inventory the four islands. A total of 85 adult specimens have been captured. All the specimens have been examined morphologically and some of them have been processed for molecular biology. All of them belong to the genera *Sergentomyia* and *Grassomyia*. Two new taxa for Science are described in the present study.

## Material and methods

**Fig. 1.**
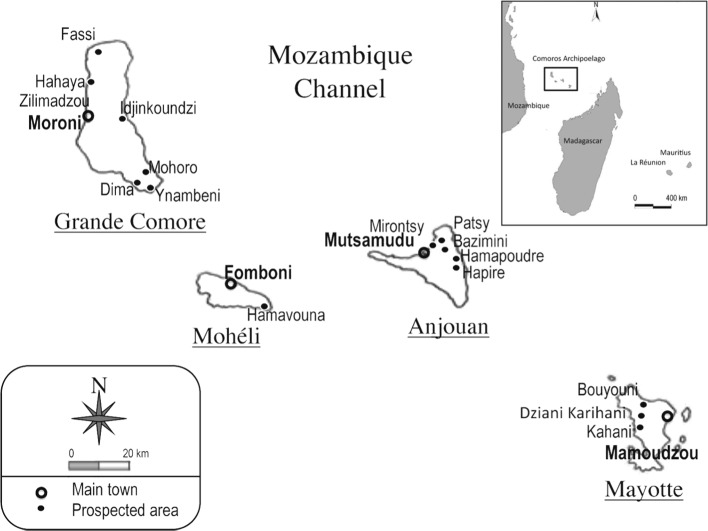
Prospected area.

Phlebotomine sandflies have been caught using CDC miniature light traps and UV miniature light traps in the four main islands of the Comoros Archipelago: Grande Comore, Mohéli, Anjouan and Mayotte (Fig. 1, Table I).

**Table I.** Sampling species.

Island	Location	Number of traps	Catching year	Specimens caught	Samples processed for molecular biology and their genbank accession numbers
Grande Comore	Fassi	6	2011	*S. goodmani comorensis*: 3 ♂	JQ421008
				JQ421009	
				JQ421010	
	Idjinkoundzi	6	2011		
	Hahaya	6	2011	*S. goodmani comorensis* : 1 ♀	JQ421012
	Ynambeni	6	2011	*S. pessoni* n. sp. : 2 ♀	JQ421019
				JQ421020	
	Mohoro	6	2011	*S. goodmani comorensis* : 1 ♀	JQ421011
	Dima	2	2011	*S. goodmani comorensis* : 1 ♀	
	Zilimadzou		2003	*S. goodmani comorensis* : 39 ♂ & 35 ♀	JQ421014
				JQ421017	
				JQ421018	

Anjouan	Patsy	1	2011		
	Mirontsy	6	2011		
	Mutsamudu	6	2011		
	Hamapoudre	6	2011	*S. goodmani comorensis* : 1 ♀	JQ421013
	Hapira	6	2011		
	Bazimini	6	2011		

Moheli	Hamavouna		2007	*Grassomyia* sp. : 2 ♀	JQ421015
					JQ421016

Mayotte	Lake of Karihani	9	2011		
	Village of Kahani	6	2011		
	Village of Bouyouni	8	2011		
	Governor’s Home	8	2011		

Madagascar	Namoroka		2002	*S. namo*	EU143774
					EU143775
				*S.goodmani*	JQ421002
					JQ421005
	Ankarana		2003	*S. sclerosiphon*	EU143777
					EU143778
					EU143780
					EU143781
					EU143783
					EU143786
					EU143787
					EU143788
					EU143789
					EU143790
				*S. goodmani*	JQ434695
				*S. anka*	EU143779
					EU143782
					EU143784
					EU143785
	Ankiliefatra		2003	*S. goodmani*	JQ421006
					JQ421003
					JQ421007
					JQ421004

The archipelago of the Comoros is located at the northern end of the Mozambique Channel in the Indian Ocean, halfway between the coasts of Madagascar and the African continent. The volcanic chain of the Comoros appears to be a “hot spot trace”. The Comoros are made up of volcanic rocks, primarily undersaturated alkali olivine basalts. Phonolitic and small volumes of trachytic lavas have also been reported (Pavlovsky & de Saint-Ours, 1953; Flower, 1973; Emerick & Duncan, 1982). The volcanic rocks have been differentiated into three volcanic phases, in which basaltic lavas prevail. Scorias and puzzolanic tuffs have been reported from Grande Comore, Mohéli and Anjouan. Phonolitic and trachytic rocks occur on Mayotte (Pavlovsky & de Saint-Ours, 1953). On the geo-chronological plan, the latest information on the age of the three islands is respectively as follows: 1.49, 0.48, 0.36 millions years (My) for Mayotte, Mohéli and Anjouan (Armstrong, 1972: Emerick & Duncan, 1982, 1983: Nougier *et al.*, 1986) and 0.13 My for Grande Comore (Emerick & Duncan, 1982, 1983).

The catching sites, including mainly caves, are detailed below.

1 – In Grande Comore:

- Fassi cave (Mitsamiouli): a hole about 15 m deep, which divides into two long passages. One is about 10 m long and is of archaeological interest, the other probably leads to the sea;
- Hahaya cave (Hamanvou): next to the runway at Hahaya airport;
- Ynambeni cave (Nioumagama): a hole about 10 m deep, dividing into two parts along the main access on about 100 meters;
- Idjinkondzi cave (Dimani), about 5 m long;
- Ceiling of Dima primary school (Nioumagama);
- Under the main mosk of the village of Mohoro (Itsahidi);
- District of Zilimadzou (Moroni), in the east border of the city.

2 – In Anjouan:

- Hamapoudre cave (Mromagi – Bambao), about 50 m long at the bottom of which lies a pond of about 4 m diameter;
- Hapire cave (Limbi): it is more like an excavation with a rocky slope of about 30 m high next to a 10 m waterfall;
- Bazimini cave (Bazimini): a hole about 5 m deep;
- Primary school of Mirontsy (Mirontsy - Mutsamudu);
- Ceiling of a social center of about 50 m × 20 m × 10 m, a hard brick building covered by a corrugated iron roof which is a shelter for little bats;
- Next to the University of Patsy.

3 – In Mohéli: Hamavouna, 20 m above sea level (a.s.l.), at the top of a steep slope at the base of a vertical stone cliff in which ten strange horizontal holes seem to have carved, 2 m deep and 10 to 30 cm diameter.

4 – In Mayotte: Lake Karihani, Village of Kahani, Village of Bouyouni and the House of the Governor.

When available, some specimens from Madagascar have been added to this sampling (Table I). Those of *Vattieromyia* refer to Depaquit *et al.* (2008) and those of *S. goodmani* have been specially processed in the present study. Sandflies were stored in 96 % ethanol. The head and genitalia were cut off in a drop of ethanol, cleared in boiling Marc-André solution, and mounted between slide and cover slide for species identification. The body related to the specimen was stored dried in a vial at - 20 °C before DNA extraction. All the specimens were observed utilizing a BX50 microscope and measured using the Perfect Image software (Aries Company, Chatillon, France) and a video camera connected to the microscope. Drawings have been done using the *camera lucida* installed on the microscope. Some specimens have been remounted in Canada balsam after complete processing of washing, dehydration in baths of ethanol 70 to 100, then beech creosote.

Genomic DNA was extracted from the thorax, wings, legs and abdomen of individual sandflies using the QIAmp DNA Mini Kit (Qiagen, Germany) following the manufacturer’s instructions, modified by crushing the sandfly tissues with a piston pellet (Treff, Switzerland), and using an elution volume of 200 μl, as detailed in (Depaquit *et al.,* 2004a). All the mtDNA amplifications were performed in a 50 μl volume using 5 μl of extracted DNA solution and 50 pmol of each of the primers. The PCR mix contained (final concentrations) 10 mM Tris HCl (pH 8.3), 1.5 mM MgCl2, 50 mM KCl, 0.01 % Triton X 100, 200 μM dNTP each base, and 1.25 units of 5 prime *Taq* polymerase (Eppendorf, Germany). The cycle begins with an initial denaturation step at 94 °C for 3 min and finishes by a final extension at 68 °C for 10 min. Amplification of a fragment of cytochrome B gene has been done by using the primers N1N-PDR: (5’-CA(T/C) ATT CAA CC(A/T) GAA TGA TA-3’) and C3B-PDR: (5’-GGT A(C/ T)(A/T) TTG CCT CGA (T/A)TT CG(T/A) TAT GA-3’) following Esseghir *et al.* (1997): five cycles of (denaturation at 94 °C for 30 s, annealing at 40 °C for 60 s and extension at 68 °C for 60 s), followed by 35 cycles of (denaturation at 94 °C for 60 s, annealing at 44 °C for 60 s and extension at 68 °C for 60 s). Amplicons were analysed by electrophoresis in 1.5 % agarose gel containing ethidium bromide. Direct sequencing in both directions was performed using the primers used for DNA amplification. The correction of sequences is done using Pregap and Gap softwares included in the Staden Package (Bonfield & Staden, 1996).

Molecular analyses are based on the sequence alignment performed using the ClustalW routine included in the bioedit version 5 software (Hall, 1999) and checked by eye. According to the objective of this study, which is not a phylogenetical analysis, a Neighbor-Joining (NJ) analysis was performed using MEGA 5 software (Tamura *et al.*, 2011), with the Kimura- 2 parameter model and using uniform rates among sites.

## Results

A total of 85 Phlebotomine sandflies has been caught, on Grande Comore, Mohéli and Anjouan (82, two and one, respectively). No sandflies have been caught in Mayotte (Table I). Most of the specimens (81 specimens) belong to the subgenus *Rondanomyia* and appear to be a subspecies of *Sergentomyia goodmani*. Two female specimens of the subgenus *Vattieromyia* belong to a new species described below. Moreover, two specimens of *Grassomyia* sp. have been caught in Mohéli.

### *Sergentomyia pessoni* n. sp. Depaquit, Randrianambinintsoa & Léger

Genus *Sergentomyia* Rondani & Berté, *in* Rondani, 1840

Subgenus *Vattieromyia* Depaquit, Léger & Robert, 2008 Species *Sergentomyia pessoni* n. sp.

The authors Depaquit, Randrianambinintsoa & Léger are responsible for satisfying the criteria of availability of the name *Sergentomyia pessoni* and should be cited as the sole authority of these taxa, according to the Article 50(1) of the lnternational Code of Zoological Nomenclature, 4^th^ edition, 2000.

**Fig. 2.**
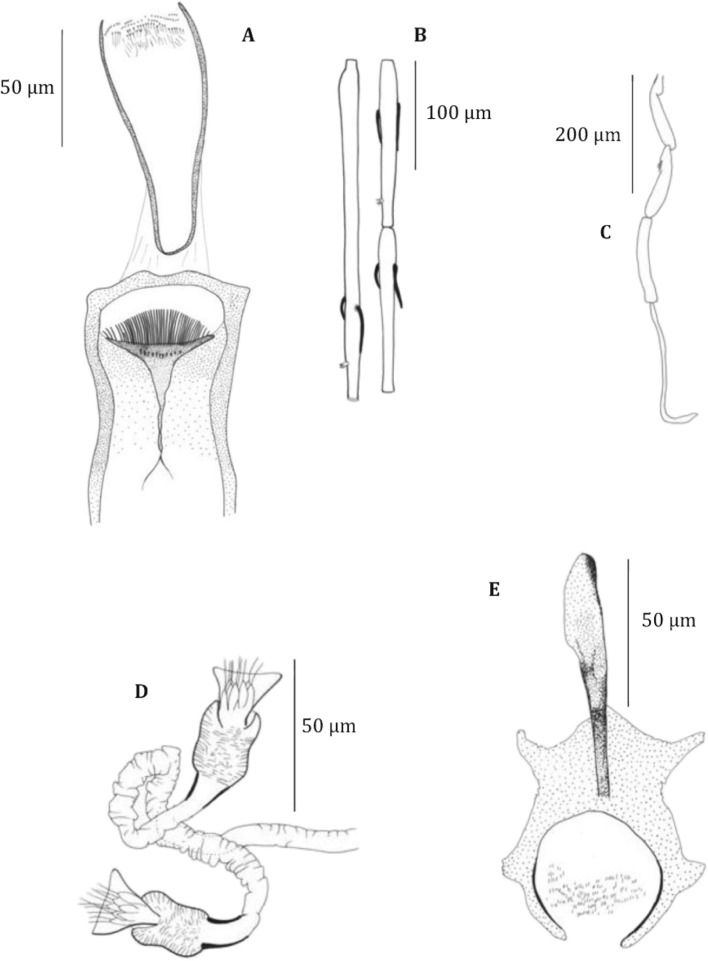
*S. pessoni* n. sp. female. A: pharynx and cibarium; B: antennal segments III, IV and V; C: palp; D: spermathecae; E: furca.

• Female (Fig. 2, Table II)

**Table II.** Descriptive statistics related to morphometry; for each character.

	Female						Male	
Character	*S. pessoni n*. sp.	S. anka*	S. namo*	S. sclerosiphon*	S. goodmani**	*S. goodmani comorensis* ssp. nov. n = 27	S. goodmani**	*S. goodmani comorensis* ssp. nov. n = 39
Labrum	189–191	170–190		158–204	150–170	156–184 (171.93 ± 9.15)	110–140	118–147 (133.21 ± 6.57)
AIII	289–297	177–204	210–280	214–300	110–130	119–158 (128.57 ± 12.49)	104–132	102–131 (118.27 ± 7.48)
AIV	145–156	94–111	100–140	105–145	64–70	61–79 (68.98 ± 5.68)	64–75	64–76 (69.21 ± 4.19)
AV	142–149	95–111	104–140	108–140	66–78	58–87 (71.44 ± 7.98)	74–80	65–76 (71.89 ± 3.15)
AIII / labrum	1.53–1.55	1.18–1.35	1.18–1.35	1.27–1.55	0.7–0.8	0.69–0.80 (0.73 ± 0.05)	0.9	0.75–1.02 (0.89 ± 0.06)
AIII / AIV + AV	0.97–1	< 1	1	1		0.88–0.92 (0.91 ± 0.04)		
Wing length	1,600–1,692	1,500–1,808		1,628–1,886	1,320	1,236–1,433 (1,349.79 ± 85.46)	1,280	1,044–1,184 (1,136.27 ± 37.31)
Wing width	475–505	401–460	430–491	410–534	400	320–418 (371.86 ± 33.98)	330	223–309 (281.36 ± 21.78)
a	493–434	259–333	326–384	251–405	200	101 -218 (177.63 ± 46.88)	200	93–150 (120.11 ± 20.95)
p	242–254	323–343	297–354	312–389	250	264–378 (304.95 ± 44.36)	220	206–273 (240.95 ± 27.86)
ô	254–331	113–339	159–211	91–247	80	11–82 (54.03 ± 30.41)		17–36 (25.31 ± 9.46)
Y	250–264	244–254	245–342	235–315	400	205–292 (256.40 ± 36.13)	90	226–238 (232.05 ± 4.50)
n	48–65	135–172	123–188	111–209		109–177 (135.05 ± 31.96)		82–120 (101.85 ± 16.97)
Wing length/width	3.35–3.37	3.74–3.93	3.56–4.13	3.53–4.15	3.30	3.39–3.87 (3.64 ± 0.16)		3.82–4.69 (4.05 ± 0.20)
Wing width/y	1.9	1.64–1.81	1.40–1.80	1.40–1.79	1.00	1.61–1.26 (1.49 ± 0.14)		2.58–3.70 (3.08 ± 0.52)
Number of cibarial teeth	44–45	35–43	48–55	20–29	16–27	15–21 (16.93 ± 1.77)	150	127–149 (139.75 ± 7.83)
Coxite length CL							15	4–6 (4.60 ± 0.68)
Number of internal setae of the coxite style length							65	56–72 (65.63 ± 4.21)
Aedeagus length							70–90	58–79 (65.81 ± 6.85)
Genital filaments							250–380	331–409 (360.11 ± 25.80)
Genital pump							75–104	81–112 (90.60 ± 9.54)
Genital filaments/ genital pump							3.57–4.24	3.73–4.68 (4.04 ± 0.29)

The minimum and maximum values are indicated on the first line whereas the mean value ± standard error are indicated on the second, in brackets. Measurments are in µm.

* from Depaquit *et al.* (2008);

** from Léger *et al.* (2005).

The description is based on the two specimens caught at Ynambeni, Grande Comore.

Head

Interocular suture almost complete.

Cibarial armature with:

- a posterior line with 44 to 45 teeth. The median ones are longer than the lateral ones. They are arched dorsoventrally;
- a line of 11 to 12 V-shaped anterior denticles. Pigment patch large, with a triangular anterior extension.

Pharyngeal armature made up of small teeth pointed forward, in concentric rows.

Palpal formula: 1, 2, 3, 4, 5. Newstead spines spatulated on the third palpal segment.

Antennal formula: 2/III-X, 1/XI-XV, or 2/III-XI-1/XIIIXV. Ascoids don’t reach the next articulation.

A III = 289–297 μm. A IV = 145–156 μm. A V = 142–149 μm. A III more or less equal to A IV + A V; A III/labrum = 1.53–1.55.

Thorax

Mesanepisternum without setae.

Wing: length = 1,600–1,692 μm; width = 475–505 μm; length/width = 3,35–3,37; a = 493–434 μm; b = 242–254 μm; d = 254–331 μm; g = 250–264 μm; width/g = 1.9; P = 48–65 μm.

Spermathecae

Typical of the subgenus *Vattieromyia*. Short cylindrical body, slightly striated on the edges nearly echinulated, more narrow in its median part than in its proximal and distal parts. Head made up of a few spear-like elements prolonged by some filaments, surrounded by a high collar. Ducts difficult to observe, slightly folded, relatively short, joining to form a common proximal duct. Individual ducts are strongly sclerotised over a limited length next in continuation with the ducts.

Type locality: cave of Ynambeni, Grande Comore, at 11°54.466’S, 43°28.615’E and 286 m a.s.l.

• Male

Unknown.

### *Sergentomyia goodmani comorensis* n. ssp. Depaquit, Randrianambinintsoa & Léger

Genus *Sergentomyia* Rondani & Berté, *in* Rondani, 1840

Subgenus *Rondanomyia* Theodor, 1958

Species *Sergentomyia goodmani* Léger, Depaquit & Robert, 2005

Subspecies *Sergentomyia goodmani comorensis* n. ssp.

The authors Depaquit, Randrianambinintsoa & Léger are responsible for satisfying the criteria of availability of the name *Sergentomyia goodmani comorensis* and should be cited as the sole authority of these taxa, according to the Article 50(1) of the lnternational Code of Zoological Nomenclature, 4th edition, 2000.

**Fig. 3.**
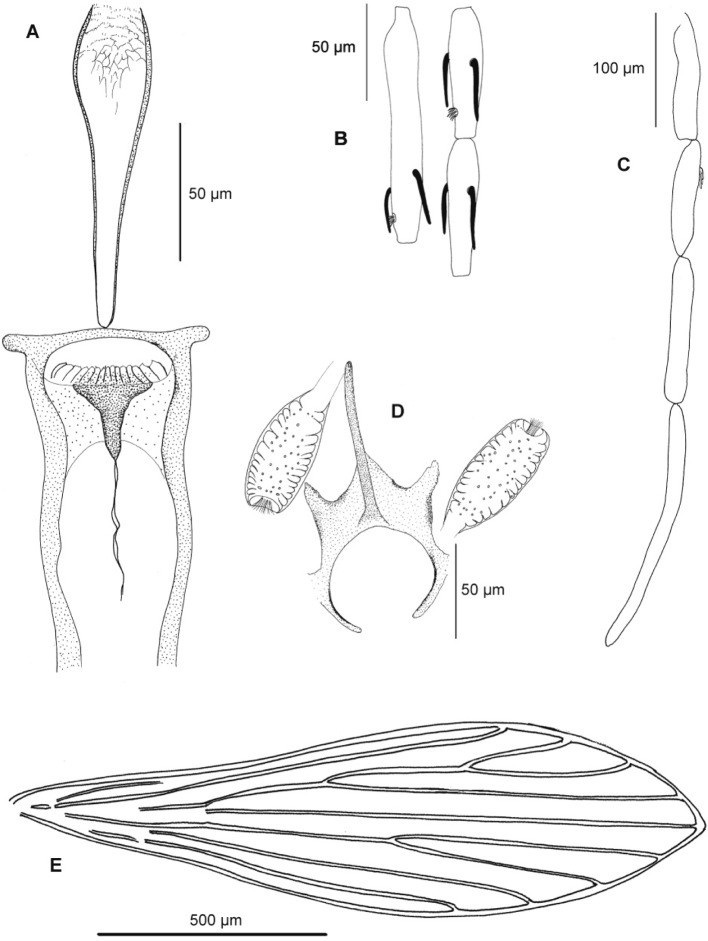
*Sergentomyia goodmani comorensis* n. ssp. female. A: pharynx and cibarium; B: antennal segments III, IV and V; C: palp; D: spermathecae and furca; E: wing.

• Female (Fig. 3, Table II)

Head

Interocular suture almost complete.

Cibarium with a line of 13 to 20 teeth like those of *Sergentomyia schwetzi*. The central ones are narrower than the lateral ones. No anterior denticles.

Large pigment patch, mushroom-like, with an anterior extension.

Pharynx narrow with i) in the back, few small teeth oriented backward displayed on concentric rounded rows, and ii) in the front, bigger teeth oriented forward.

Palpal formula: 1, 2, 3, 4, 5. Newstead spines spatulated on the third segment.

Antennal formula: 2/III-XV; short ascoids don’t reach the next articulation.

Short antennal segments: AIII = 119–158 μm; AIV = 61- 79 μm ; AV = 55–87 μm. AIII/AIV+AV = 0.88 - 0.92. Labrum = 156–184 μm.

Thorax

Mesanepisternum without setae.

Wings: length = 1,236–1,433 μm; width = 320–418 μm; α = 101–218 μm; β = 264–378 μm; δ = 11–82 μm; γ = 205–292 μm; wing width/ = 1.26–1.61; P = 109–177 μm. No spines on the legs.

Spermathecae

Typical of the subgenus *Rondanomyia*: cylindrical body, smooth, covered with many spikes oriented towards the light of the spermathecae, thereby its incomplete ringed. Knob in a pit. Ducts very difficult to observe.

**Fig. 4.**
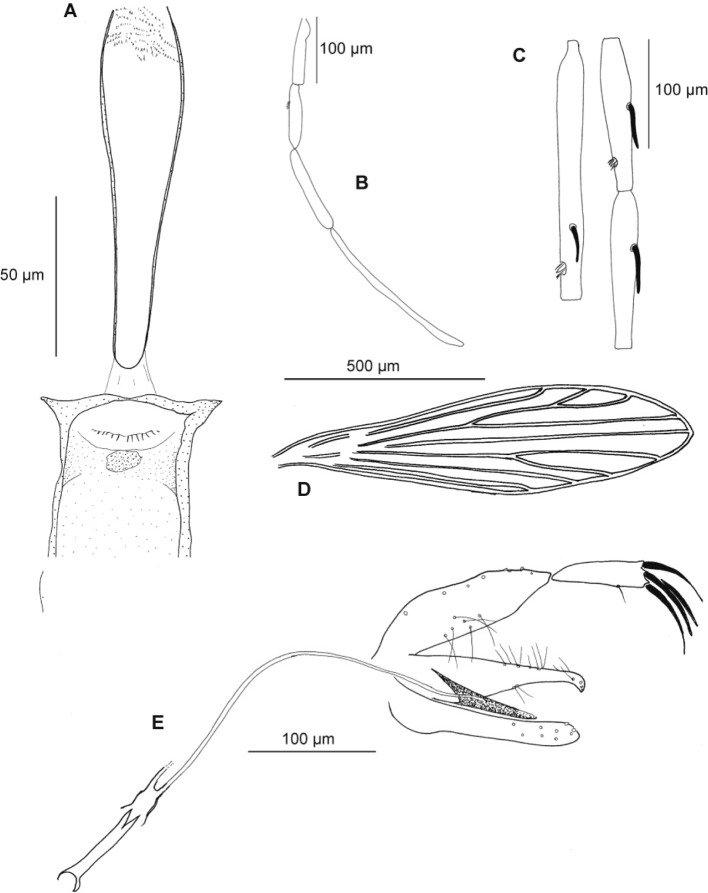
*Sergentomyia goodmani comorensis* n. ssp. male. A: cibarium and pharynx; B: palp; C: antennal segments III, IV and V; D: wing; E: genitalia.

• Male (Fig. 4, Table II)

Head

Interocular suture almost complete, similar to that of the female.

Cibarial armature with a few teeth, similar to that of *S. goodmani*. Limited pigment patch. Narrow pharynx. Pharyngeal armature less developed than that of the female. Palpal formula: 1, 2, 3, 4, 5. Newstead spines on the third palpal segment.

Antennal formula: 1/III-XII, with short ascoids; AIII = 104–132 μm ; AIV = 64–76 μm : AV = 65–76 μm. Labrum = 118–147 μm.

Thorax

Mesanepisternum without setae.

Wing: length = 1,044–1,184 μm; width = 223–309 μm; α = 93–150 μm; β = 206–273 μm; γ = 226–238 μm; width/g = 2.58–3.70; P = 82–120 μm.

No spines on the legs.

Genitalia

Closely resembling that of the specimens of Madagascar. Coxite long of 127–149 μm, exhibiting on its inner face a group of four to six disseminated setae.

Sharp style, 56 to 72 μm long with four terminal spines and an accessory seta located at the level of the distal third or quarter.

Simple paramere with a hooked extremity.

Aedeagus 58 to 79 μm long, straight, sharped and sometimes pointed at the top.

Genital filaments 331 to 409 μm long. Genital pump 81 to 112 μm long.

Genital filaments/genital pump = 3.73 to 4.68.

Type locality: Zilimandzou, Grande Comore, at 11°42.583’S, 43°14.847’E and 26 m a.s.l.

### Derivatio nominum

*S. pessoni* is dedicated to our colleague Bernard Pesson.

The subspecies comorensis refers to the geographical origin of the studied population.

The holotype and the paratype of *S. pessoni*, and the holotype and nine paratypes (including four allotypes) of *S. goodmani comorensis* have been deposited in the Muséum national d’Histoire naturelle of Paris.

### Molecular results

The sequences studied were 497 to 503, depending on species and geographical origin (Table I). An alignment made by ClustalW included in the Bioedit package, and checked by eye, includes 507 positions.

**Fig. 5.**
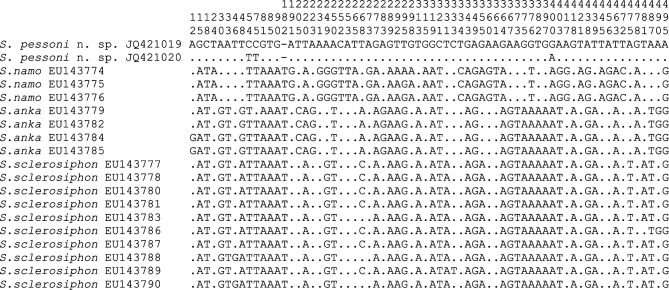
Variable positions observed within species belonging to the subgenus *Vattier omyia* of *Sergentomyia* for cytochrome b sequences.

**Fig. 6.**
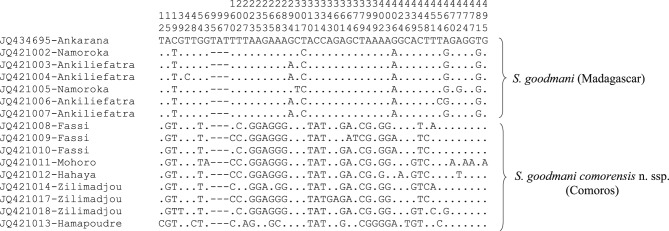
Variable positions observed between *Sergentomyia goodmani* and for cytochrome b sequences.

An analysis of the variable sites within the *Vattieromyia* shows 68 variable sites (Fig. 5). An analysis of the variable sites within the *Rondanomyia* shows 51 variable sites (Fig. 6).

The pairwise distances between and within groups are showed on Table III.

**Table III.** Pairwise genetic distances (%) between and within taxa.

	*S. goodmani madagascarensis*	*S. goodmani comorensis*	*Grassomyia*	*S.pessoni*	*S.namo*	*S.scleosiphon*	*S Pairwise distance anka within taxa*
*S. goodmani madagascarensis*							0.65
*S. goodmani comorensis*	5.78						1.83
*Grassomyia*	25.30	25.05					1.44
*S. pessoni*	25.38	25.70	16.85				0.61
*S. namo*	22.34	23.43	17.79	9.65			0.14
*S. scleosiphon*	24.55	24.59	18.22	9.25	8.71		0.29
*S. anka*	23.87	23.08	16.98	9.35	9.40	2.72	0.24

**Fig. 7.**
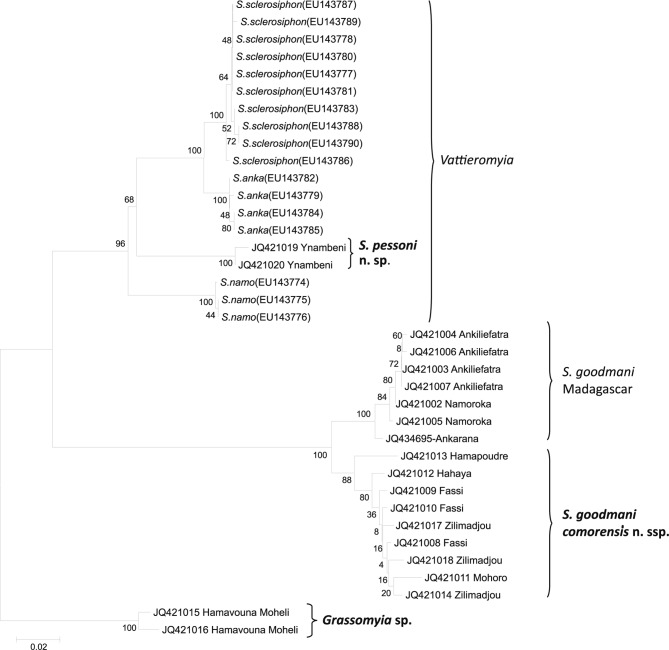
Neighbor-Joining tree based on cytochrome b sequences mtDNA. Bootstrap values indicated have been obtained after 1,000 replications.

The NJ tree based on cytochrome B sequences is given in Fig. 7. Within the *Vattieromyia*, the species *Sergentomyia sclerosiphon*, *S. namo* and *S. anka* are as strongly supported (bootstrap value: 100 %) as the two specimens caught in the Comoros islands.

Concerning the *Rondanomyia*, we observe a dichotomy between Malagasy and Comorian specimens. The Malagasy specimens are supported by 100 % bootstrap whereas Comorian specimens are supported by 88 % bootstrap. The specimen from Anjouan shows a haplotype differing by many nucleotides from the haplotypes of specimens caught in Grande Comore (Fig. 6). An additional NJ tree (data not shown) carried out without this specimen shows an increasing bootstrap value (96 %).

## Discussion

Until an expected revision of its taxonomy has been done, the genus *Sergentomyia* França & Parrot, 1920 currently includes all the species excluded from the other genera of the Old World (*Phlebotomus*, *Idiophlebotomus*, *Chinius*, *Spelaeophlebotomus*, *Grassomyia*, *Parvidens*, *Spelaeomyia* and *Demeillonius*). Concerning the *Grassomyia*, we refer to the position of Abonnenc & Léger (1976) considering this group as a genus. The genus *Sergentomyia* is presently divided without any phylogenetical argument into six subgenera: *Sergentomyia* França & Parrot, 1920, *Parrotomyia* Theodor, 1948, *Rondanomyia* Theodor, 1948, *Sintonius* Nitzulescu, 1931, *Capensomyia* Davidson, 1978 and *Vattieromyia* Depaquit, Léger & Robert 2007 (Duckhouse & Lewis, 1980; Léger & Depaquit, 1999; Depaquit, Léger & Robert, 2007). The males being difficult to identify, this classification is mainly based on the morphology of the female spermathecae. Moreover, some species are still ungrouped, due to their atypical spermathecae.

In 2007, Depaquit *et al.* defined the subgenus *Vattieromyia* by in the female i) the shape of the spermathecae and the sclerotized parts of their ducts, ii) the cibarial armature palisade-like, iii) an unusual antennal formula without ascoid on AIII. The males also have this original antennal formula shared only by *Grassomyia* spp. and *S. majungaensis*. At the light of *S. pessoni* n. sp., it appears that this species belong to the subgenus *Vattieromyia* according to the first two characters defining this group. This very original spermathecae has a more important weight than the original antennal formula. Consequently, we now exclude the antennal formula without ascoid on AIII from the definition of the subgenus *Vattieromyia*.

The morphological differences observed between the three Malagasy species belonging to the subgenus *Vattieromyia* and the two Comorian females appear to be of specific level. The females of *S. pessoni* n. sp. differ from all the Malagasy species by the presence of ascoids on the third antennal segment. Moreover, they are different from *S. sclerosiphon* by the number of cibarial teeth, from *S. namo* by the lack of cibarial ring and from *S. anka* by the shape of the cibarial armature and by the number of denticle ranges. The individualization of *S. pessoni* n. sp. is reinforced by the molecular data, also individualizing this new species.

Concerning *S. goodmani*, a strong molecular dichotomy is observed between the Malagasy specimens and the Comorian ones. However, there is little morphological divergence between Comorian and Malagasy specimens. This suggests an occurring speciation event linked with the insularity rather than a speciation fully achieved. Consequently, we consider *S. goodmani comorensis* n. ssp. as a subspecies of *S. goodmani* and not as a new species because both their molecular and morphological characters are more discreet and, linked to insularity, perfectly correspond to the concept of subspecies *sensu* Mayr *et al.* (1953). Although we are unable to test the interfecondity which could exist between Malagasy and Comorian populations. To individualize this subspecies, we consider i) in females the smaller number of cibarial teeth (15 to 21) and some morphometrical characters of the wing and the antennae and ii) in males, mainly the very small number of coxal setae in the Comorian specimens (four to six without real tuft *versus* about 15 in specimens from Madagascar).

We can’t identify the *Grassomyia* females caught during the present study at a specific level. The two specimens have been mounted for molecular biology processing (Table III) and consequently, their thoraces have been crushed for DNA extraction. The main characters used for the identification of the species of the genus *Grassomyia* are the absence/presence (and number) of setae on the mesanepimerum and the absence/presence of spines on the anterior and medium femurs (*G. dreyfussi* Parrot). According to the well developed pharyngeal armature of our specimens from Mohéli, their number of cibarial teeth (34 and 39), and to the characters revised by Abonnenc (1969, 1972), it cannot belong to *G. inermis* Theodor, 1938 (lack of pharyngeal armature, 19 to 25 cibarial teeth), or to *G. squamipleuris* Newstead, 1912 (48 to 55 cibarial teeth). Even though we have not observed the femurs of the specimens from Mohéli, the latter cannot be *G. dreyfussi* Parrot, 1933 (42 to 55 cibarial teeth and femurs with spines). Consequently, the Comorian specimens could be *G. madagascarensis* Abonnenc, 1969 (36 to 43 cibarial teeth), or *G. ghesquierei* Parrot, 1929 (27 to 37 cibarial teeth, lack of setae on the mesanepimerum). A complete revision of this group should be carried out in the future.

Nothing is known about the vectorial competence of the three species found in Comoros islands. Although no leishmaniasis cases have been reported, further studies are needed to evaluate the risk of autochtonous transmission of *Leishmania*.
